# Rapid Quantification of Bluetongue Virus-Neutralizing Antibodies Using Bioluminescent Reporter-Expressing Viruses

**DOI:** 10.3390/vaccines13111102

**Published:** 2025-10-29

**Authors:** Luis Jiménez-Cabello, Sergio Utrilla-Trigo, Eva Calvo-Pinilla, Aitor Nogales, Javier Ortego

**Affiliations:** Centro de Investigación en Sanidad Animal (CISA), Instituto Nacional de Investigación y Tecnología Agraria y Alimentaria (INIA), Valdeolmos, 28130 Madrid, Spain; sergio.utrilla@inia.csic.es (S.U.-T.); calvo.eva@inia.csic.es (E.C.-P.); nogales.aitor@inia.csic.es (A.N.); ortego@inia.csic.es (J.O.)

**Keywords:** Bluetongue virus (BTV), neutralizing antibodies, reverse genetics, reporter gene, luciferase

## Abstract

Bluetongue virus (BTV) is the causative agent of the significant livestock disease Bluetongue (BT), which causes severe economic losses associated with its considerable impact on the health and trade of ruminants. **Background/Objectives**: BTV infection and vaccination against the virus typically result in the induction of antibodies with the capacity to neutralize viral infection. Classic neutralization approaches resemble the methodology applied for neutralizing antibodies (NAbs) quantification. To improve long-standing and new-generation methodologies for the quantification of NAbs or evaluation of antivirals, we offer here the development of a new luciferase-based microneutralization approach as a proof-of-concept. **Methods**: Central to this innovative approach is the recently generated set of replication-competent reporter-expressing recombinant BTV, where the NanoLuc luciferase protein expression serves as a quantifiable readout for viral replication. After evaluating a set of heat-inactivated serum samples with neutralizing activity (measured via SNTs), these were incubated with 100 PFU of NLuc-expressing rBTV of serotype 1, 4 or 8 and Vero cells were infected with the serum–virus mixture. Then, the luminescent signal was measured at 48 h post-infection. **Results**: Using the proposed NLuc-based assay and the luminescent signal in the supernatant, we could detect neutralizing activity as soon as 48 h post-infection. Importantly, we were able to observe a strong correlation between NAbs titers measured by classic microneutralization assay and by our bioluminescent approach (BTV-1 Spearman r = 0.932901; *p*-value < 0.0001; BTV-4 Spearman r = 0.8070192; *p*-value < 0.0001; BTV-8 Spearman r = 0.9983; *p*-value < 0.0001). In addition, the NLuc-based assay displayed a serotype-specific character potentially equivalent to classic SNT methods. **Conclusions**: In summary, our reporter-based microneutralization assay provides a rapid and suitable method to quantify BTV-neutralizing antibodies in serum samples of natural hosts after vaccination or infection, with a serotype-specificity equivalent to classic SNT methods.

## 1. Introduction

Bluetongue (BT) is an arthropod-borne disease that affects wild and domestic ruminants [1,2]. The Bluetongue virus (BTV) (family *Sedoreoviridae*), which causes BT and is transmitted by Culicoides biting midges [3,4], is composed of a three-layered protein capsid harboring a double-stranded RNA (dsRNA) segmented genome [5,6,7]. The ten dsRNA segments encode for seven structural proteins (VP1–VP7) and five nonstructural proteins (NS1–NS5) [5,6,8,9,10,11,12]. Protein VP2 is the most exposed virion protein as it forms trimers located in the outer capsid layer [7]. This protein acts during the onset of cell infection through its interaction with the cellular receptor, allowing virus entry into the host cell [13,14,15]. Consequently, VP2 is the main target of virus-neutralizing antibodies (NAbs) and the determinant of virus serotype [16,17,18]. To date, more than 30 serotypes of BTV have been described, and due to the high genetic variability of protein VP2 among serotypes (especially in antigenically relevant regions [19,20]), neutralization among BTV serotypes is scarce [21,22,23]. As conventional vaccine approaches and most next-generation vaccines base their protective potential against BTV on the presentation/expression of protein VP2 [18,24,25], quantification of NAbs serves as a measure of vaccine efficacy. In this sense, a classic serum neutralization test (SNT) is considered as the gold standard for the detection and quantification of NAbs responses in BTV-exposed and -vaccinated animals [7,26]. Nonetheless, NAbs quantification using a plaque reduction neutralization test (PRNT) and a microneutralization assay are labor-intensive and time-consuming as they involve a plate incubation period of five days, and may lead to misinterpreted or inconsistent results [26,27].

Alternative methodologies for NAbs quantification have been developed against different viruses, such as pestiviruses, SARS-CoV-2, Rift Valley fever virus or Influenza virus [28,29,30,31,32]. These approaches exploit reverse genetics (RG) systems to generate fluorescent or luminescent viruses that allow us to avoid the utilization of fixative agents and offer an easier and more homogenous measurement than classical cell-based approaches, which rely on a reduction in virus-induced cytopathic effect (CPE). Similarly, reporter viruses have been confirmed as reliable tools to facilitate antiviral drug testing [33,34] as they enhance the efficiency and accuracy of antiviral compound identification compared to traditional methods. In this sense, genetically engineered reporter viruses permit rapid screening of large compound libraries, which boosts high-throughput drug screening [35,36,37].

Diverse RG systems exist for orbiviruses such as BTV, African Horse Sickness virus (AHSV) or Epizootic Hemorrhagic Disease virus (EHDV) [38,39,40,41]. Recently, we developed a whole-plasmid-based RG system for the rescue of recombinant BTV [42]. By using this RG system, we successfully recovered a recombinant BTV expressing reporter luminescent (NanoLuc luciferase, NLuc) gene [42]. Thus, this proof-of-concept study aims to demonstrate the feasibility of a methodology for the rapid and easy quantification of serotype-specific NAbs using luminescent reporter BTV as an alternative to a classical SNT. In addition, we also study the possibilities of these NLuc-expressing BTVs for the in vitro evaluation of antivirals against this viral pathogen.

## 2. Materials and Methods

### 2.1. Cell and Viruses

Green monkey kidney cells (Vero) (ATCC catalog no. CCL-81) were grown in Dulbecco’s modified Eagle’s medium (DMEM) supplemented with 5% heat-inactivated fetal bovine serum (FBS) and 1% PSG (penicillin, 100 units/mL; streptomycin 100 µg/mL; l-glutamine, 2 mM) at 37 °C in air enriched with 5% CO_2_.

BTV serotype 1 (ALG2006/01) (BTV-1), BTV serotype 4 (SPA2004/02), BTV serotype 8 (BEL/2006), rBTV-1/NLuc, rBTV-4/Luc and rBTV-8/NLuc were used in the experiments. Generation of rBTV-1/NLuc, rBTV-4/Luc and rBTV-8/NLuc has previously been described [42]. Vero cells were used for the generation of virus stocks and virus titration was performed via plaque assay, as previously described [42]. This research was performed in the Animal Health Research Center (CISA, INIA-CSIC), a biological security facility (BSL3).

### 2.2. Microneutralization Assay

Two-fold or four-fold dilutions of heat-inactivated sheep, cow or mice sera (56 °C for 30 min) were incubated with 100 PFU of BTV-1, BTV-4 or BTV-8 for 1 h at 37 °C and 4 °C O/N. Vero cells (96-well plate format, 5 × 10^4^ cells/well, triplicates) were then infected with the serum–virus mixture for 1 h at 37 °C, 5% CO_2_. Then, the serum–virus mixture was removed and 150 µL of fresh growth medium were added and incubated for 5 days at 37 °C, 5% CO_2_. Thereafter, Vero cells were fixed with paraformaldehyde 4% and visualized with 2% crystal violet-PBS. The neutralization titer (named neutralization dilution 50, ND_50_, to differentiate from the neutralization titer calculated by NLuc signal) is defined as the reciprocal of the highest dilution that shows ≥50% inhibition of virus infectivity.

### 2.3. NLuc-Based Microneutralization Assays

NLuc microneutralization assays were performed as previously described [43,44]. Two-fold or four-fold dilutions of heat-inactivated sheep, cow or mice sera (56 °C for 30 min) were incubated with 100 PFU of rBTV-1/NLuc, rBTV-4/NLuc, or rBTV-8/NLuc for 1 h at 37 °C and at 4 °C, O/N. Vero cells (96-well plate format, 5 × 10^4^ cells/well, triplicates) were then infected with the serum–virus mixture for 1 h at 37 °C, 5% CO_2_. Then, the serum–virus mixture was removed and 150 µL of fresh growth medium were added and incubated for 72 h at 37 °C. At 48 or 72 h post-infection (h.p.i.), NLuc activity in the cell culture supernatants was quantified using Nano-Glo luciferase substrate (Promega, Madrid, Spain) and a FLUOstar Omega microplate reader (BMG Labtech, Ortenberg, Germany). The luminescent values of virus-infected cells in the absence of antibody were used to calculate 100% viral infection. Cells in the absence of viral infection were used to calculate the luminescence background. Triplicate wells were used to calculate the average and SD of neutralization using Microsoft Excel software. The 50% neutralization titer (NT_50_) was determined by use of a sigmoidal dose–response curve (GraphPad Prism, v8.0.1 software). GraphPad Prism software was used to perform Spearman correlation analysis between NAbs titers obtained by classic or NLuc-based microneutralization assays. Bland–Altman plots were made with GraphPad Prism software.

### 2.4. Antiviral Assays

Monolayers of Vero cells in 96-well plate format (5 × 10^4^ cells/well, triplicates) were infected with 100 PFU of rBTV-1/NLuc. After 90 min of viral adsorption, the medium was replaced with 150 µL of medium (containing only 2% FBS) supplemented with two-fold serial dilutions (starting concentration, 125 μM) of ATA, and the cells were then incubated at 37 °C, 5% CO_2_. At 48 h.p.i., NLuc activity in tissue culture supernatants was measured using Nano-Glo luciferase substrate (Promega) and FLUOstar Omega microplate reader (BMG Labtech). The luminescent values of virus-infected cells in the absence of ATA were used to calculate 100% viral infection. Cells in the absence of viral infection were used to calculate the luminescence background. Triplicate wells were used to calculate the average and SD of the inhibition using Microsoft Excel software. The 50% inhibition concentration (IC_50_) was determined by the use of a sigmoidal dose–response curve (GraphPad Prism, v8.0.1 software).

## 3. Results

### 3.1. Use of Luminescent BTVs for Determination of BTV-NAb Titers

**Figure 1:**
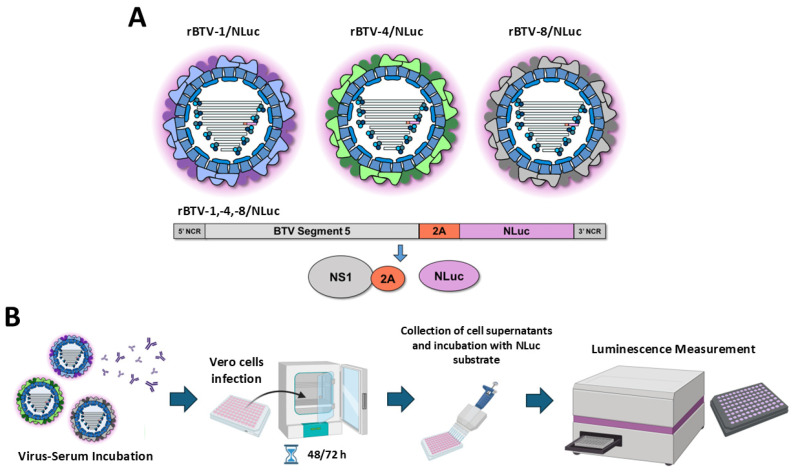
(**A**) Representation of the recombinant BTV/Luc viruses used in this study. NLuc reporter gene was included as fusion proteins by means of a 2A picornavirus peptide fused to the 3′ end of segment 5 of BTV-1. (**B**) Schematic representation of the methodological procedure to measure virus infection by means of luminescent signal in supernatants of Vero cells infected with reporter viruses.

By implementing a whole-plasmid RG system for BTV, we previously engineered and characterized recombinant BTVs of serotypes 1, 4 and 8 expressing a reporter luminescent (NLuc) gene [42]. The reporter gene was fused to the C-terminal end of segment 5 (S5) of BTV by means of the porcine teschovirus-1 (PTV-1) 2A cleavage sequence (Figure 1A). S5 encodes non-structural protein 1 (NS1) and, consequently, expression of the reporter gene only occurs during the replicative cycle of BTV. Reporter-expressing BTVs were used to study the dynamics of viral infection in vitro and in vivo, and the expression of the NLuc reporter gene constituted a valid surrogate for virus replication [42]. Now, we have used these luminescent recombinant BTVs of serotype 1 (rBTV-1/NLuc), serotype 4 (rBTV-4/NLuc) and serotype 8 (rBTV-8/NLuc) for the rapid quantification of NAbs against BTV-1, BTV-4 and BTV-8, respectively, using an NLuc-based microneutralization assay (Figure 1B), as has previously been performed for other RNA viruses [30,31,32].

An SNT to determine antibody-mediated virus inhibition requires the use of secondary approaches to detect the presence of the virus. To overcome this additional step and validate our NLuc-based microneutralization assay, rBTV-1/NLuc, rBTV-4/NLuc and rBTV-8/NLuc viruses were used to evaluate the neutralizing activity of serum samples obtained from BTV-exposed or -vaccinated animals. First, we tested if virus neutralization could be tracked by luminescent signal using two serum samples from cow (obtained after experimental infection, Neutralizing Dilution 50 (ND_50_) = 1:160) or sheep (obtained after immunization with a recombinant vaccine [45], ND_50_ = 1:240) reactive to BTV-1 or BTV-4, respectively, and a mouse serum hyperimmune against BTV-8 (ND_50_ = 1:400), as we lacked a natural BTV host serum reactive to BTV-8. To do so, two-fold dilutions of these heat-inactivated serum samples were pre-incubated with 100 PFU of rBTV-1/NLuc, rBTV-4/NLuc and rBTV-8/NLuc viruses, and Vero cells were infected with the virus–serum mixture. As a control, we included a non-reactive serum against BTV, as well as mock-infected cells and viruses in the absence of antibodies as internal controls. At 48 or 72 h.p.i., supernatants were collected, and the luminescent signal was measured.

**Figure 2:**
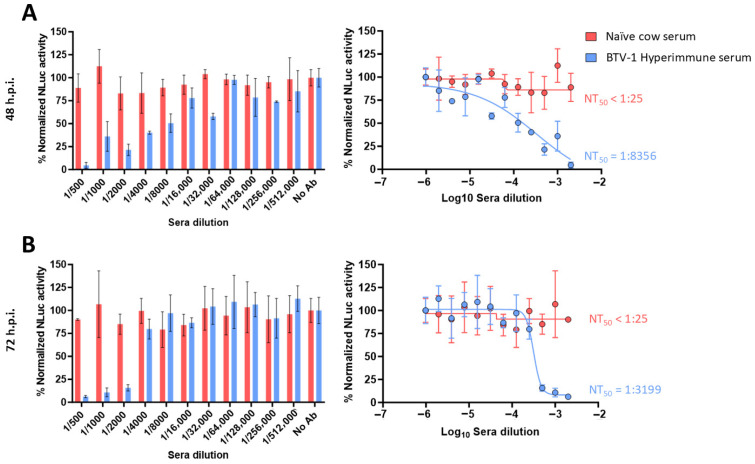
A luciferase-based microneutralization assay for the identification of NAbs against BTV-1. Vero cells were infected (100 PFU) with rBTV-1/NLuc pre-incubated with 2-fold serial dilutions (starting dilution of 1:500) of a BTV-1-exposed cow serum sample. At 48 (**A**) or 72 (**B**) h.p.i., NLuc activity in culture supernatants was quantified using a microplate reader, and the percentage of inhibition was calculated using sigmoidal dose–response curves. A negative serum obtained from a naïve cow was used as the control. Mock-infected cells and viruses in the absence of antibodies were used as internal controls. The percentage of luminescent activity was normalized to Vero cell infection with 100 PFU of rBTV-1/NLuc in the absence of serum (100% of luminescent activity) and Vero cells in the absence of virus (0% of luminescent activity). Data show the means ± SD of the results determined in triplicate. The starting dilution was established considering preliminary data.

**Figure 3:**
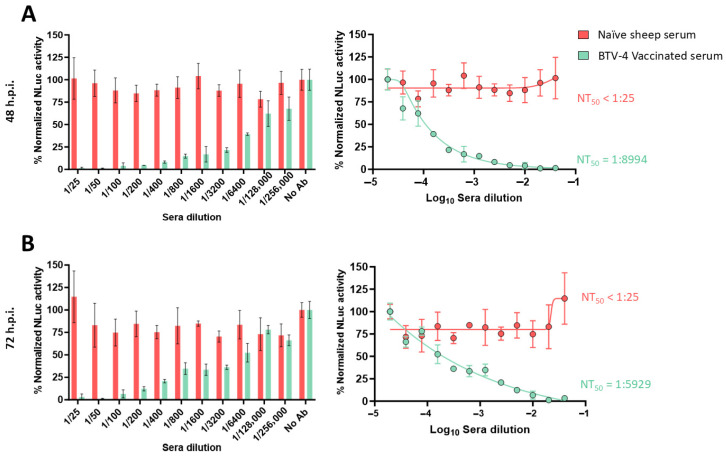
A luciferase-based microneutralization assay for the identification of NAbs against BTV-4. Vero cells were infected (100 PFU) with rBTV-4/NLuc pre-incubated with 2-fold serial dilutions (starting dilution of 1:25) of an inactivated-BTV-4-vaccinated sheep serum sample. At 48 (**A**) or 72 (**B**) h.p.i., NLuc activity in culture supernatants was quantified using a microplate reader, and the percentage of inhibition was calculated using sigmoidal dose–response curves. A negative serum obtained from a naïve sheep was used as the control. Mock-infected cells and viruses in the absence of antibodies were used as internal controls. The percentage of luminescent activity was normalized to Vero cell infection with 100 PFU of rBTV-4/NLuc in the absence of serum (100% of luminescent activity) and Vero cells in the absence of virus (0% of luminescent activity). Data show the means ± SD of the results determined in triplicate. The starting dilution was established considering preliminary data.

**Figure 4:**
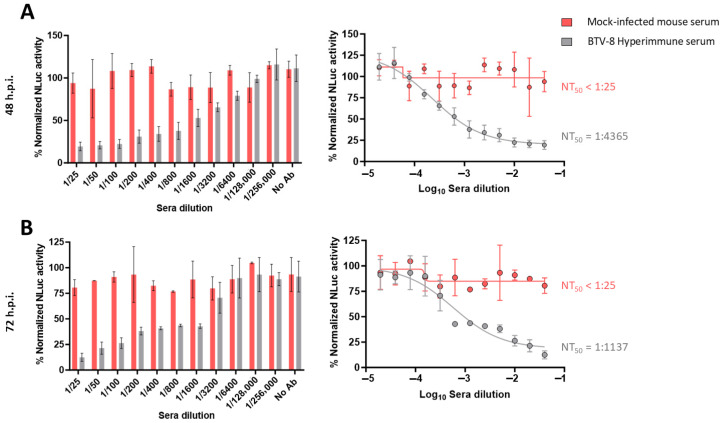
A luciferase-based microneutralization assay for the identification of NAbs against BTV-8. Vero cells were infected (100 PFU) with rBTV-8/NLuc pre-incubated with 2-fold serial dilutions (starting dilution of 1:25) of a BTV-8 hyperimmune mouse sera sample. At 48 (**A**) or 72 (**B**) h.p.i., NLuc activity in culture supernatants was quantified using a microplate reader, and the percentage of inhibition was calculated using sigmoidal dose–response curves. A negative serum (from a mock-infected mouse) was used as a control. Mock-infected cells and viruses in the absence of antibodies were used as internal controls. The percentage of luminescent activity was normalized to Vero cell infection with 100 PFU of rBTV-8/NLuc in the absence of serum (100% of luminescent activity) and Vero cells in the absence of virus (0% of luminescent activity). Data show the means ± SD of the results determined for triplicates. The starting dilution was established considering preliminary data.

In our luciferase-based microneutralization assay, higher NLuc activity indicated higher viral replication and, therefore, lower neutralization levels. The negative serum sample did not neutralize rBTV-1/NLuc, rBTV-4/NLuc or rBTV-8/NLuc, as luminescent signals equivalent to the virus-infected cells without serum (internal control) were observed at 48 and 72 h.p.i. even at the lower serum dilution evaluated (Figure 2, Figure 3 and Figure 4). In contrast, we observed that the serum obtained from a BTV-1-infected cow induced a dilution-dependent reduction in the percentage of NLuc activity at 48 h.p.i. (Figure 2A) and 72 h.p.i. (Figure 2B). Similarly, we observed that the tested serum that was reactive to BTV-4, obtained from a BTV-4-vaccinated sheep [45], also neutralized the reporter rBTV-4/NLuc at 48 h.p.i. (Figure 3A) and 72 h.p.i. (Figure 3B), as indicated by the antibody-dilution-dependent detection of luminescent activity. Luminescent signals from supernatants of Vero cells infected with rBTV-8/NLuc also indicated that the mouse serum hyperimmune against BTV-8 neutralized the reporter virus at both times post-infection (Figure 4A,B).

It is worth noting that these sera against BTV-1, BTV-4 or BTV-8 did not show cross-neutralizing activity against the heterologous serotypes evaluated here, as determined via a classic SNT. Importantly, the three tested serum samples did not exhibit a serum-induced reduction in NLuc activity when their neutralizing activity was assessed against heterologous NLuc-expressing BTV serotypes, which mildly reflects the serotype-specificity of our NLuc-based microneutralization test.

Using the sigmoidal dose–response curves, we could calculate the neutralizing titer 50 (NT_50_) as a measure of virus neutralization based on the NLuc signal. The NT_50_ values of the cow and sheep sera against rBTV-1/NLuc or rBTV-4/NLuc, respectively, were higher at 48 h.p.i. (cow serum NT_50_ = 1:8356; sheep serum NT_50_ = 1:8994) than at 72 h.p.i. (cow serum NT_50_ = 1:3214; sheep serum NT_50_ = 1:5929), which is intrinsically related to less viral replication (and lower viral protein expression) at early times of infection. In the case of rBTV-8/NLuc, we could estimate NT_50_ values of 1:4365 (48 h.p.i) and 1:1137 (72 h.p.i.) against BTV-8. Despite the differences between the NT_50_ estimated through NLuc measurement at two and three days of Vero cells infection, our luciferase-based microneutralization assay was able to determine the presence of homologous NAbs as early as 48 h.p.i. Therefore, we selected the measurement of NLuc activity at 48 h.p.i. to confirm its suitability as a rapid alternative method to quantify NAbs in further studies.

### 3.2. NAbs Quantification by Luminescence Activity Correlates with NAbs Titer Determined via Classic SNT

**Figure 5:**
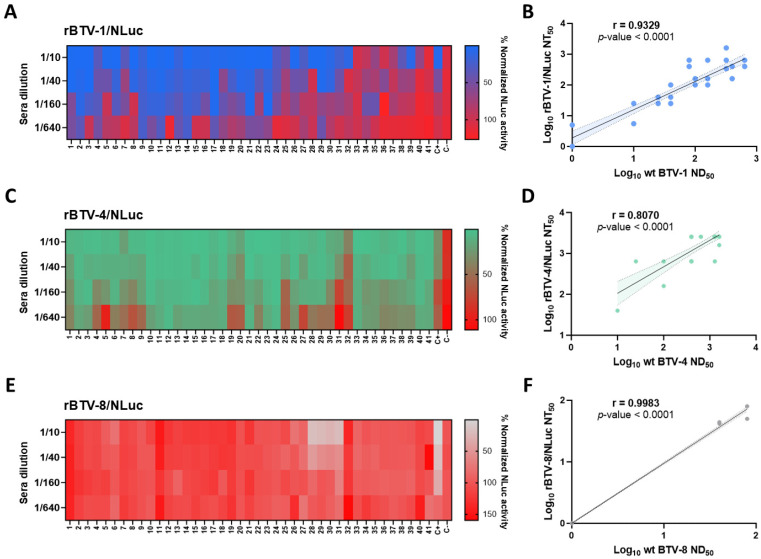
Identification of NAbs against BTV in a collection of serum samples. Vero cells were infected (100 PFU) with rBTV-1/NLuc (**A**), rBTV-4/NLuc (**C**) or rBTV-8/NLuc (**E**) pre-incubated with 4-fold serial dilutions (starting dilution of 1:10) of sera samples. At 48 h.p.i., NLuc activity was quantified using a microplate reader. A negative sheep serum was used as the control. Mock-infected cells and viruses in the absence of antibodies were used as internal controls. The percentage of neutralization was normalized to Vero cell infection with 100 PFU of each recombinant BTV in the absence of antibodies (100% of luminescent activity) and Vero cells in the absence of virus (0% of luminescent activity). (**B**,**D**,**F**) Correlation between neutralization titers values obtained via SNT or NLuc signal against wild-type BTV-1 and rBTV-1/NLuc (**B**), wild-type BTV-4 and rBTV-4/NLuc (**D**) or wild-type BTV-8 and rBTV-8/NLuc (**F**), as calculated by Spearman’s rank order correlation. Confidence intervals of 95% are indicated by gray dotted lines. ND_50_ refers to NAbs titer quantified by classic microneutralization assay. NT_50_ refers to NAbs titer quantified by NLuc-based assay.

Despite the fact that luminescent signal in the supernatants of reporter BTV-infected cells indicated virus neutralization in the previous experiment, we could not establish a correlation between a classic SNT and an NLuc-based assay as we evaluated one positive sample for each BTV serotype. Therefore, we applied this rapid methodology to conduct a screening of NAbs in a set of BTV-positive sheep sera samples kindly provided by the Central Veterinary Laboratory (LCV Algete, Spain). For BTV-1-positive serum samples (estimated via classic microneutralization test, see Table 1), we detected NT_50_ values above the lower dilution tested (1:10), with some of them reaching titers of 1:640 (Figure 5A). BTV-1-negative serum samples (ID: 31, 33, 34, 35, 36, 37, 40 and 41) displayed high percentages of NLuc activity at the lowest dilution evaluated (1:10), and therefore did not show evidence of neutralization against rBTV-1/NLuc (Figure 5A).

It is worth noting that most serum samples were collected from BTV-4-exposed animals, as all the animals (except for sheep 32) had high titers of NAbs against this BTV serotype (estimated via classic microneutralization test, see Table 2). Interestingly, we observed that all the serum samples, except for the serum from sheep 32, showed NT_50_ values of over 1:40 against rBTV-4/NLuc (Figure 5C).

The set of sera showed neutralizing activity against BTV-1 and BTV-4, which is in line with the co-circulation of both serotypes in some regions of Spain. However, there is no evidence of BTV-8 circulation in 2023 [46], when the samples were collected. As expected, most serum samples did not show evidence of neutralizing activity against BTV-8 by either luminescent signal determination or a classic SNT (Figure 5E). However, sera from sheep 28, 29, 30 and 31 showed NT_50_ values between 40 and 80 (Figure 5E). This could imply that our NLuc-based microneutralization test is not completely serotype-specific. Nonetheless, these four sera showed neutralizing capacity, as measured using a classic microneutralization test (see Table 3), which might indicate that our bioluminescent assay could have an equivalent sensibility to traditional methodologies.

**Figure 6:**
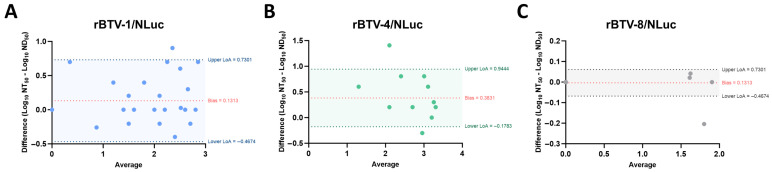
Bland–Altman plots showing the relationship between the measurement of neutralizing activity via classic microneutralization assay (ND_50_) and NLuc-based microneutralization assay (NT_50_) for (**A**) BTV-1, (**B**) BTV-4 and (**C**) BTV-8. The mean Log_10_ value of each pair of measures is plotted on the X-axis and the difference between each pair of measures is plotted as Log_10_ values on the Y-axis. The bias is indicated as a red dotted line. Upper and lower limits of agreement (LoA) were calculated as ± 1.96 times the standard deviation of the differences and are indicated as colored dotted lines. The values of the bias and the upper and lower LoA are indicated in each plot.

To assess whether there is correlation between classic neutralization and the proposed bioluminescent assays, we determined the neutralization titers of the whole set of field sera against non-reporter wild-type BTV of serotype 1, 4 and 8 via classic microneutralization assay. NAbs titers of field sera against wild-type BTV-1, BTV-4 and BTV-8 and NT_50_ values against rBTV-1/NLuc, rBTV-4/NLuc and rBTV-8/NLuc are gathered in Table 1, Table 2 and Table 3, respectively. Notably, we observed a strong correlation between neutralization titers against BTV-1 and rBTV-1/NLuc (Spearman r = 0.9329; 95% confidence intervals (CIs) = 0.8770–0.9639; *p*-value < 0.0001) (Figure 5B), and between BTV-4 and rBTV-4/NLuc (Spearman r = 0.8070; 95% CI = 0.6643–0.8930; *p*-value < 0.0001) (Figure 5D). Additionally, the data provided by our NLuc-based assay using rBTV-8/NLuc correlated with data gathered via classic microneutralization assay (Spearman r = 0.9983; 95% CI = 0.9967–0.9991; *p*-value < 0.0001) (Figure 5F). We also assessed the agreement between these two quantitative measurement methods for each serotype using Bland–Altman plots (Figure 6). On average, we observed that the NLuc-based microneutralization assay resulted in slightly higher neutralization values compared to the classic microneutralization assay, except for BTV-8 (BTV serotype 1 bias = 0.1313, upper limit of agreement (LoA) = 0.7301, lower LoA = −0.4674; BTV serotype 4 bias = 0.3831, upper LoA = 0.9444, lower LoA = −0.1783; BTV serotype 8 bias = −0.004352, upper LoA = 0.06106, lower LoA = −0.06797). Nonetheless, results involving BTV serotype 8 need to be confirmed more robustly by using a wider set of serum samples reactive to BTV-8 in further experiments. Overall, these data indicate the potential feasibility of luminescent recombinant viruses for the detection and quantification of serotype-specific NAbs.

### 3.3. NLuc-Based Microneutralization Assay for Assessment of Antiviral Efficacy Against BTV

**Figure 7:**
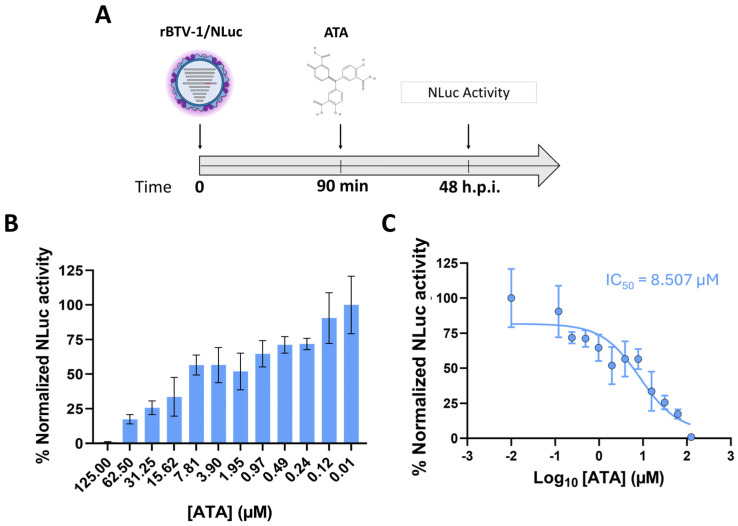
A luciferase-based assay for the identification of compounds with antiviral activity against BTV. (**A**) Vero cells were infected (100 PFU) with rBTV-1/NLuc and incubated with serial two-fold dilutions (starting concentration 125 μM) of ATA. At 48 h.p.i., (**B**) NLuc activity was quantified using a microplate reader, and (**C**) the IC_50_ value was calculated using sigmoidal dose–response curves. Mock-infected cells and virus in the absence of drugs were used as internal controls. Percentage of inhibition was normalized to Vero cell infection with rBTV-1/Venus in the absence of drugs (100% of luminescent activity) and Vero cells in the absence of virus (0% of luminescent activity). The percentage of inhibition was normalized to infection in the absence of drugs. Data show the means ± SD of the results determined in triplicate.

Considering the usefulness of luminescent reporter BTVs for quantification of NAbs against BTV, we performed a first approach to evaluate the utility of this methodology for the evaluation of antivirals against BTV. Previously, we had determined that the polyanionic aromatic compound aurintricarboxylic acid (ATA) has inhibitory activity against Orbivirus replication in vitro [47]. To test if the therapeutic activity of ATA could be evaluated using NLuc measurement, Vero cells were infected with rBTV-1/NLuc. After 90 min of viral absorption, the virus inoculum was replaced with infection medium containing two-fold serial dilutions (starting concentration of 125 μM) of ATA. At 48 h.p.i, tissue culture supernatants were collected, NLuc activity was measured, and the 50% inhibitory concentration (IC_50_) was calculated (Figure 7A). Treatment of rBTV-1/Nluc-infected cells with ATA induced a dose-dependent reduction in NLuc activity in the supernatant at 48 h.p.i. (Figure 7B). These data coincide with the ATA-induced dose-dependent inhibition of viral replication previously observed for BTV [47]. Indeed, the IC_50_ value obtained using rBTV-1/NLuc via measurement of NLuc activity (IC_50_ = 8.507 µM) is similar to that reported for wild-type BTV-4 (IC_50_ = 2.7 µM) obtained by classic plaque assay in Vero cells [47]. These data indicate that luminescent reporter BTVs can also be an effective and rapid option for screening compounds with antiviral activity.

## 4. Discussion

Providing the possibility to monitor BTV infection in real time is advantageous to study the biology of the virus, to evaluate the presence of NAbs or the efficacy of antiviral compounds, and to test the effectiveness of novel vaccination strategies. However, studying BTV usually involves secondary experimental techniques to detect the virus in infected cells, which limits the study of the virus to some extent. Considering recent outbreaks involving novel serotypes of BTV and the complexity of its epidemiology, which involves insect vector transmission and a plethora of serologically non-related serotypes (specially at cross-neutralization level) [3,4,21,22,23,48,49,50], it is of paramount importance to establish robust and rapid methodologies for determining NAb activity in BTV-affected areas, as well as to rapidly and more accurately assess the efficacy of novel vaccination strategies. Recently, we took advantage of RG techniques for BTV [39,51,52,53,54,55] to generate new replication-competent reporter rBTVs expressing the bioluminescent NanoLuc protein [42]. These rBTVs expressing the NLuc luminescent reporter gene have allowed us to easily track viral infections in cultured cells and mice without the need for traditional secondary methods [42]. Therefore, we used these newly developed NLuc-expressing BTVs to design an easy, fast and precise methodology to quantify neutralizing activity against the virus as proof-of-concept.

Cloning of the NLuc reporter gene fused to segment 5 of BTV, which encodes protein NS1, permits the study of different aspects of the biology of BTV, because the expression of the reporter gene is a measurement of viral replication and spread [42]. Considering that rBTV-driven NLuc expression correlated with virus replication [42], we tested whether NLuc-expressing rBTV could be used to quantify NAbs via a luciferase-based microneutralization assay. By using well-known serum samples with high neutralizing activity against BTV-1, BTV-4 or BTV-8, we demonstrated that our luminescence-based approach has the capacity to detect the presence of NAbs against BTV. To further validate our results, we tested a collection of field serum samples provided by the Central Veterinary Laboratory (LCV Algete, Spain), the European reference laboratory for BTV. Our NLuc-based microneutralization assay was able to quantify the NAbs titers against either BTV-1, BTV-4 or BTV-8, demonstrating the capacity of this methodology for the rapid detection of NAbs, even in complex field samples. Importantly, the outcome obtained by our NLuc-based assay strongly correlated with data obtained by a classic SNT, indicating the applicability of the proposed methodology. It is highly important to develop rapid and precise assays to detect the presence of NAbs in multiple samples to control outbreaks more efficiently, to take rapid and rigorous decisions to limit the propagation of the disease, and to better understand the epidemiological situation. In this regard, our NLuc-based microneutralization assay quantifies the highly sensitive luciferase NLuc, providing an accurate measurement of neutralizing activity compared to classic methods for NAbs quantification, whose outcome can vary depending on technical procedures. Furthermore, traditional SNTs are based directly on viral replication and spread, which usually takes around five days and requires the use of toxic fixative agents such as formaldehyde. Conversely, we were able to quantify NAbs in serum samples as early as 48 h post-infection in absence of fixation. Therefore, our luminescent microneutralization assay provides equivalent results to classic SNTs, is user-friendly and time-effective, and reduces the likelihood of inaccuracy.

Bioluminescence can be detected and quantified very soon after BTV infection, as it depends on viral protein expression [42]. Thus, reliable determination of neutralizing activity could be performed as early as 48 h.p.i., which is one of the main strengths of the proposed NLuc-based methodology. Nonetheless, quantification of the neutralizing activity could also be conducted at 72 h.p.i. Despite three days post-infection being near the incubation period that classic SNT approaches require, it still offers advantages such as lack of fixative agents and reduced probability of inaccuracy. Nevertheless, extended incubation periods much longer than 48 h.p.i. might lead to the accumulation of NLuc in rBTV/NLuc-infected cell supernatants and a cumulative bioluminescence signal, inducing potential underestimation of neutralizing activity. Indeed, if we observe the 72 h.p.i. dose–response curves of the positive serum samples against rBTV-1/NLuc or rBTV-8/NLuc, the bioluminescent activity abruptly escalated in just one dilution from less than 25% to more than 75% of normalized luminescence, and from 40% to 70% of normalized luminescence, respectively. In contrast, NLuc measurement at 48 h.p.i. displayed a marked serum-dilution effect. This was one of the main reasons why the set of serum samples for the quantification of neutralization was tested at 48 h.p.i. instead of 72 h.p.i. Still, quantification at 72 h.p.i. might be adequate depending on the fitness of rBTV in vitro, which could have implications on inter-laboratory standardization. The generation of NLuc-expressing recombinant BTV of different serotypes depends on the substitution of segments 2 and 6 that encode proteins VP2 and VP5, respectively, while maintaining a backbone of another serotype (in our case, BTV-1) [42]. As described before [56], the insertion of heterologous proteins VP2 and VP5 can lead to chimeric recombinant BTVs that exhibit restricted replicative capacity in vitro (cloning of the reporter gene NLuc also impacts viral fitness [42]). Therefore, weakened replication at 48 h.p.i. could lead to lower viral protein expression and overestimated neutralizing activity, whereas quantification at 72 h.p.i. might be a preferred option due to higher NLuc expression. Anyway, if this is the case, correlation between classic microneutralization assay and NLuc-based quantification at 72 h.p.i. should be demonstrated.

Due to the wide plethora of different serotypes of BTV and the complexity of cross-neutralizing relationships among them [20,22], serotype-specificity must be a requisite of any serological assay that aims to quantify NAbs against BTV. Like classic SNTs, where viral replication-induced CPE defines the results, our NLuc-based approach relies on viral protein expression and, in extension, viral replication. In consequence, it should have the capacity to offer a serotype-specific quantification of Nabs, as in traditional approaches. In this sense, our luminescent microneutralization assay provided differential quantification of serotype-specific NAbs against BTV in the whole set of serum samples evaluated, matching the detection of serotype-specific NAbs determined by a traditional SNT approach. In addition, whereas most serum samples could not neutralize BTV-8, we detected mild neutralizing activity against this serotype in four serum samples when applying our NLuc-based assay. Considering that serotype 8 of BTV did not circulate in the Spanish areas where BTV-1 and BTV-4 co-circulate at the time the sera were collected, it might indicate that our novel approach does not offer reliable serotype-specificity. Nonetheless, these samples were also positive for neutralizing activity against BTV-8 measured using a classic SNT approach, which may be due to cross-neutralization between these BTV serotypes and could indicate that the detection of NAbs against BTV-8 was not a shortcoming of our bioluminescent approach. Previous studies have described serological relationships between serotypes circulating in Europe (1, 2, 4, 8 and 9) and partial cross-neutralization between these serotypes [57,58]. In any case, our data demonstrates the capability of this NLuc-based approach to detect serotype-specific antibodies from BTV-1 and BTV-4-infected or -vaccinated animals with an equivalent accuracy to traditional SNTs and in a faster way. One of the main limitations of the present study is the absence of a larger set of serum samples (especially in the case of BTV-8, as we could not include a significant number of sera reactive to this serotype). Thus, further research should be conducted with a wider set of serum samples to assure the serotype-specific character of this innovative methodology.

Recently, suitable alternatives to tedious SNT methods for determining serotype have been being developed. Enzyme-linked immunosorbent assays (ELISAs) able to detect BTV-1- or BTV-4-specific antibodies from BTV-infected or -vaccinated animals using recombinant BTV VP2 protein have been optimized [59,60]. The ELISA assay eliminates the use of cell cultures, live virus and several days of incubation. Although this assay is an attractive alternative diagnostic method to SNT methods, it does not avoid the problem of cross-reaction in sera from animals infected with genetically closely related serotypes of BTV either.

Vaccination is the most effective strategy to prevent BTV infection and spread [24]. However, this prophylactic method takes between weeks and months to show protection [61,62,63]. Therefore, alternative approaches to confront BTV epizootic events that complement vaccination programs are of interest. Despite the fact that few antivirals have been tested for the treatment of infections caused by BTV or other orbiviruses [47,64,65], identification of novel compounds with antiviral activity against them could contribute to controlling these viral diseases, reducing their spread as well as their economic impact. For this reason, we previously demonstrated that the compound ATA has a potent broad-spectrum antiviral activity, effectively inhibiting BTV and AHSV replication in vitro across mammalian and insect cells in a concentration-dependent manner [47]. Previously, ATA antiviral efficacy in vitro was measured via classic plaque assay, taking up to eight days to obtain results. Now, we have developed an NLuc-based assay for the rapid identification of antivirals using NLuc-expressing rBTV and confirmed its usefulness and reliability. This proof-of-concept demonstrated that our NLuc-based assessment of ATA efficacy against BTV was able to quantify a similar IC_50_ than that measured by classic approaches as soon as 48 h.p.i. in the absence of fixative agents. Other antivirals against BTV have been assessed by quantitative PCR [65], which implies the need for additional steps such as RNA extraction and cDNA synthesis, increasing the costs and time needed compared to NLuc measurement. The lack of in vivo efficacy against BTVs of ATA or other antivirals such as fluvastatin [47,64] highlights the need for further research to develop or identify effective treatments for orbivirus infections. Although this is a first approach to assess antiviral activity using bioluminescent reporter BTV, real-time monitoring of BTV infection dynamics according to NLuc expression could potentially optimize high-throughput screening (HTS) of novel compounds, enhancing the efficiency and accuracy of antiviral compound identification against BTV. HTS is an efficient methodology that optimizes the identification of antiviral compounds. HTS usually employs single-target or multitarget cell-free approaches that rely on biochemical assays, but cell-based assays are also an alternative, including the use of live viruses [66]. Phenotypic cell-based assays are based on a reduction in virus-induced cytopathic effect (which takes at least 72 h.p.i.) as a surrogate of viral replication. An HTS of antiviral compounds against BTV was based on a luminescent cell viability assay [67]. Measuring viral load in HTS requires techniques that include secondary approaches such as immunofluorescence assays or the AlphaLISA immunoassay, which show high robustness and reproducibility [35,68]. In contrast, reporter viruses such as our luminescent rBTV are ideal alternatives, as NLuc expression and bioluminescence signal would serve as a rapid, valid, cost-effective and straightforward readout of viral replication. Further research should be performed to confirm the adaptability of these reporter-expressing viruses to HTS assays.

While reporter viruses can significantly enhance HTS assays or optimize the serological quantification of neutralizing activity, challenges such as potential virulence alterations and reporter gene stability are of concern [69]. In the case of replication-competent, reporter-expressing rBTV, these two limitations should be considered too. Expression of reporter genes from the viral genome usually results in viral attenuation in vivo [43,70,71,72]. In this sense, insertion of the NLuc gene in the S5 of BTV led to mild attenuation compared to wild-type BTV in the BTV-susceptible IFNAR (−/−) mice [42]. Cloning of NLuc in BTV S5 also seemed to slightly reduce viral replication in vitro. However, if we consider the strong correlation between the NAbs titers quantified using either wild-type or NLuc-expressing viruses, this mildly weakened replication in vitro does not seem to be a significant issue to consider. Another potential limitation is the stability of the foreign sequence after serial passage of the virus. In the case of BTV, this stability issue can be related to the BTV strain, the reporter gene used or its location within the genome of BTV [72]. Indeed, insertion of the Venus Fluorescent Protein gene into S5 is more problematic in terms of virulence and stability than cloning of the NLuc gene, which could be related to the small size of the NLuc protein [69,73]. Luminescent rBTVs used in this work are completely stable up to four consecutive passages in cell culture [42]. Therefore, no issues should exist when considering the stability of the NLuc reporter gene in virus working stocks. Anyway, if the methodology presented here is used, it is paramount to monitor and confirm the expression of the NLuc reporter gene from reporter-expressing rBTV stocks, guaranteeing the uniform expression of the NLuc gene from the viral preparation. Ongoing research is needed to develop more stable viral constructs that enhance stability of reporter-expressing recombinant BTVs.

## 5. Conclusions

Considering the implementation of RG for other orbiviruses such as AHSV and EHDV [38,40,74,75], this proof-of-concept presents valuable insights towards the development of easier, accurate and cost- and time-effective methodologies for orbivirus NAbs quantification, with potential applicability in orbivirus-affected regions. Additionally, the functionality of using reporter-expressing viruses opens new possibilities for researchers to develop innovative cell-based assays for the quantification of NAbs, the identification of antiviral compounds or the study of host factors involved in BTV infection. Although we have demonstrated the practical viability of our NLuc-based methodology, further research will be conducted to provide more robust evidence of its utility and its potential use as a diagnostic tool.

## Figures and Tables

**Table 1:** Neutralization titer values of field sera against recombinant BTV-1 and rBTV-1/NLuc.

Sheep ID	BTV-1 (ND_50_)	rBTV-1/NLuc (NT_50_)	Sheep ID	BTV-1 (ND_50_)	rBTV-1/NLuc (NT_50_)
1	100	100	22	320	340
2	320	640	23	160	160
3	100	160	24	100	100
4	320	1600	25	25	25
5	25	40	26	25	25
6	100	100	27	25	25
7	400	160	28	40	25
8	160	100	29	100	100
9	80	640	30	100	100
10	640	400	31	10	25
11	320	640	32	25	40
12	640	400	33	0	0
13	320	640	34	10	25
14	160	640	35	0	5.5
15	80	400	36	0	0
16	160	160	37	0	5.5
17	640	640	38	320	340
18	40	100	39	40	40
19	40	40	40	10	5.5
20	400	400	41	0	0
21	640	400			

ND_50_ refers to NAbs titer quantified by classic microneutralization assay. NT_50_ refers to NAbs titer quantified by NLuc-based assay. ND_50_ and NT_50_ values under the lowest dilution applied were assigned a value of 0. ND_50_ and NT_50_ were determined in triplicate and the mean value is shown. NT_50_ CV = 1.1576. ND_50_ CV = 1.3069.

**Table 2:** Neutralization titer values of field sera against recombinant BTV-4 and rBTV-4/NLuc.

Sheep ID	BTV-4 (ND_50_)	rBTV-4/NLuc (NT_50_)	Sheep ID	BTV-4 (ND_50_)	rBTV-4/NLuc (NT_50_)
1	1600	1600	22	400	2560
2	1280	2560	23	1600	2560
3	1280	2560	24	1600	2560
4	400	640	25	100	640
5	400	640	26	400	2560
6	640	2560	27	400	640
7	400	2560	28	640	2560
8	400	640	29	400	2560
9	400	640	30	400	2560
10	1280	2560	31	100	160
11	1280	2560	32	10	40
12	1280	2560	33	1280	2560
13	1280	2560	34	1280	2560
14	1280	2560	35	1280	2560
15	1280	2560	36	1280	2560
16	1280	2560	37	1280	2560
17	1280	2560	38	1280	2560
18	1280	2560	39	1280	2560
19	1280	640	40	1280	2560
20	25	640	41	1280	2560
21	1280	2560			

ND_50_ refers to NAbs titer quantified by classic microneutralization assay. NT_50_ refers to NAbs titer quantified by NLuc-based assay. ND_50_ and NT_50_ were determined in triplicate and the mean value is shown. NT_50_ CV = 0.5343. ND_50_ CV = 0.4347.

**Table 3:** Neutralization titer values of field sera against recombinant BTV-8 and rBTV-8/NLuc.

Sheep ID	BTV-8 (ND_50_)	rBTV-8/NLuc (NT_50_)	Sheep ID	BTV-8 (ND_50_)	rBTV-8/NLuc (NT_50_)
1	0	0	22	0	0
2	0	0	23	0	0
3	0	0	24	0	0
4	0	0	25	0	0
5	0	0	26	0	0
6	0	0	27	0	0
7	0	0	28	80	50
8	0	0	29	40	42
9	0	0	30	80	80
10	0	0	31	40	44
11	0	0	32	0	0
12	0	0	33	0	0
13	0	0	34	0	0
14	0	0	35	0	0
15	0	0	36	0	0
16	0	0	37	0	0
17	0	0	38	0	0
18	0	0	39	0	0
19	0	0	40	0	0
20	0	0	41	0	0
21	0	0			

ND_50_ refers to NAbs titer quantified by classic microneutralization assay. NT_50_ refers to NAbs titer quantified by NLuc-based assay. ND_50_ and NT_50_ values under the lowest dilution applied were assigned a value of 0. ND_50_ and NT_50_ were determined in triplicate and the mean value is shown. NT50 CV = 2.7854. ND50 CV = 2.6966.

## Data Availability

The data presented in this study are available on request from the corresponding author.
